# Case report of a family with hereditary inclusion body myopathy with *VCP* gene variant and literature review

**DOI:** 10.3389/fneur.2023.1290960

**Published:** 2023-12-11

**Authors:** Greta Asadauskaitė, Ramunė Vilimienė, Vytautas Augustinavičius, Birutė Burnytė

**Affiliations:** ^1^Faculty of Medicine, Vilnius University, Vilnius, Lithuania; ^2^Faculty of Medicine, Institute of Clinical Medicine, Vilnius University, Vilnius, Lithuania; ^3^Center of Radiology and Nuclear Medicine, Vilnius University Hospital Santaros Klinikos, Vilnius, Lithuania; ^4^Faculty of Medicine, Institute of Biomedical Sciences, Vilnius University, Vilnius, Lithuania

**Keywords:** VCP gene, VCP related disease, inclusion body myopathy, multisystem proteinopathy, degenerative disease

## Abstract

**Background:**

Missense *VCP* gene variants lead to a disruption in protein homeostasis causing a spectrum of progressive degenerative diseases. Myopathy is the most frequent manifestation characterized by slowly progressing weakness of proximal and distal limb muscles. We present a family with myopathy due to c.277C > T variant in *VCP* gene.

**Case presentation:**

The patient‘s phenotype includes symmetrical muscle wasting and weakness in the proximal parts of the limbs and axial muscles, a wide based gait, lordotic posture, positive Gowers’ sign, mild calf enlargement, impaired mobility, elevated CK, and myopathy in proximal limb muscles. Whole body MRI revealed fatty replacement, predominantly affecting right vastus intermedius and medialis, gastrocnemius and soleus in calf, abdomen wall and lumbar muscles. Next-generation sequencing analysis revealed a pathogenic heterozygous variant c.277C > T (p.(Arg93Cys)) in exon 3 of the *VCP* gene. Segregation analysis showed that the detected variant is inherited from the affected father who developed symptoms at 60.

**Conclusion:**

The patients described experienced muscle wasting and weakness in the proximal and distal parts of the limbs which is a common finding in VCP related disease. Nevertheless, the patient has distinguishing features, such as high CK levels, early onset of the disease, and rapid mobility decline.

## 1 Introduction

Valosin-containing protein (VCP) related disease is a rare, autosomal dominant, multisystem proteinopathy characterized by inclusion body myopathy (IBM), Paget disease of bone (PDB) and frontotemporal dementia (FTD), affecting around 90%, 28–42%, and 14–30% of patients accordingly (1, 2). Other manifestations include amyotrophic lateral sclerosis (ALS), Alzheimer’s disease, Parkinson’s disease, Charcot–Marie-Tooth type 2 disease and complex hereditary spastic paraplegia (3–6).

VCP related disease has been associated with heterozygous missense variants in *VCP* gene. VCP belongs to the ATPases associated with diverse cellular Activities (AAA+) family, which uses ATP for protein remodeling. Each subunit contains N-terminal binding domain and two ATPase domains, D1 and D2 (7, 8). VCP is involved in a variety of cellular activities such as cell cycle control, organelle biogenesis and elimination, cellular signaling, membrane fusion, transcription, regulation of autophagy and protein degradation (8, 9). Missense variants at the NTD-D1 interface of the VCP are thought to cause a disruption in protein homeostasis causing a spectrum of progressive degenerative diseases (7, 10, 11).

In this paper we report patient and his father with c.277C > T [p.(Arg93Cys)] variant in *VCP* gene, presenting with IBM. Patient‘s and his father’s phenotype is compared with phenotypes reported in literature.

## 2 Case description

Proband is a 49-year-old male. At the age of 38, the patient reported difficulties in standing-up from a sitting position. Symptoms showed a slow but progressive worsening. He noticed upper and lower limb weakness and difficulty in daily activities such as carrying a child and stumbling. At the age of 49, the patient had difficulties in walking unaided, climbing up the stairs, and standing up from sitting and lying positions (Table 1).

**Table 1:** Patients’ timelines.

Proband					
Onset of symptoms	Neurological examination: symmetrical muscle wasting of proximal parts of the limbs	CK 1237 U/L	Whole body bone scintigraphy showed no abnormalities	CK 1309 U/L	Progression of muscle weakness, impaired mobility
38 y.o	46 y.o			47 y.o	49 y.o
Proband‘s paternal uncle					
Onset of symptoms			Died (suddenly)		
40 y.o			59 y.o		
Proband‘s father					
Onset of symptoms	Started using crutches		CK 119 U/L	Whole body bone scintigraphy showed no abnormalities	
60 y.o	68 y.o		69 y.o	70 y.o	

CK, creatine kinase.

Neurological examination at the age of 46 revealed symmetric atrophy and weakness of limb proximal muscle. Shoulder abduction and adduction were 4/5 grades (MRC-scale) on both sides. Elbow flexion and extension 4/5 bilaterally. Wrist and fingers flexion and extension were normal. Hip flexion was 3/5, hip extension - 4/5 bilaterally. Knee flexion and extension were 3/5 grades bilaterally. Ankle plantar flexion was 4/5 and dorsiflexion - 4/5 on both sides. We observed weakness of axial muscles predominantly affecting the paraspinal and abdominal muscles. The patient showed a wide based gait with a lordotic posture. Positive Gowers’ sign and mild enlargement of the calves was also noted. Tendon reflexes, sensory examination, and cranial nerve examination were normal. Cognitive testing did not reveal any frontal lobe and other cognitive abnormalities. During 4 years of follow up the muscle weakness slowly progressed. We noticed the muscle strength deterioration in the distal parts of the upper limbs. A slight asymmetry of muscle strength appeared with the right limbs being more affected. The patient can walk unaided a few meters and uses walking sticks most of the time. We did not find any cognitive deterioration. A nerve conduction study at the age of 46 did not show any abnormalities. Needle electromyography revealed myopathic changes without spontaneous activity. The myopathic pattern was more prominent in the proximal parts of the limbs. His creatine kinase (CK) level at the age of 46 was 1,237 U/L (reference range: 25–195 U/L), it increased to 1,309 U/L after a year. Whole body bone scintigraphy showed no abnormalities characteristic of Paget’s disease.

**Figure 1:**
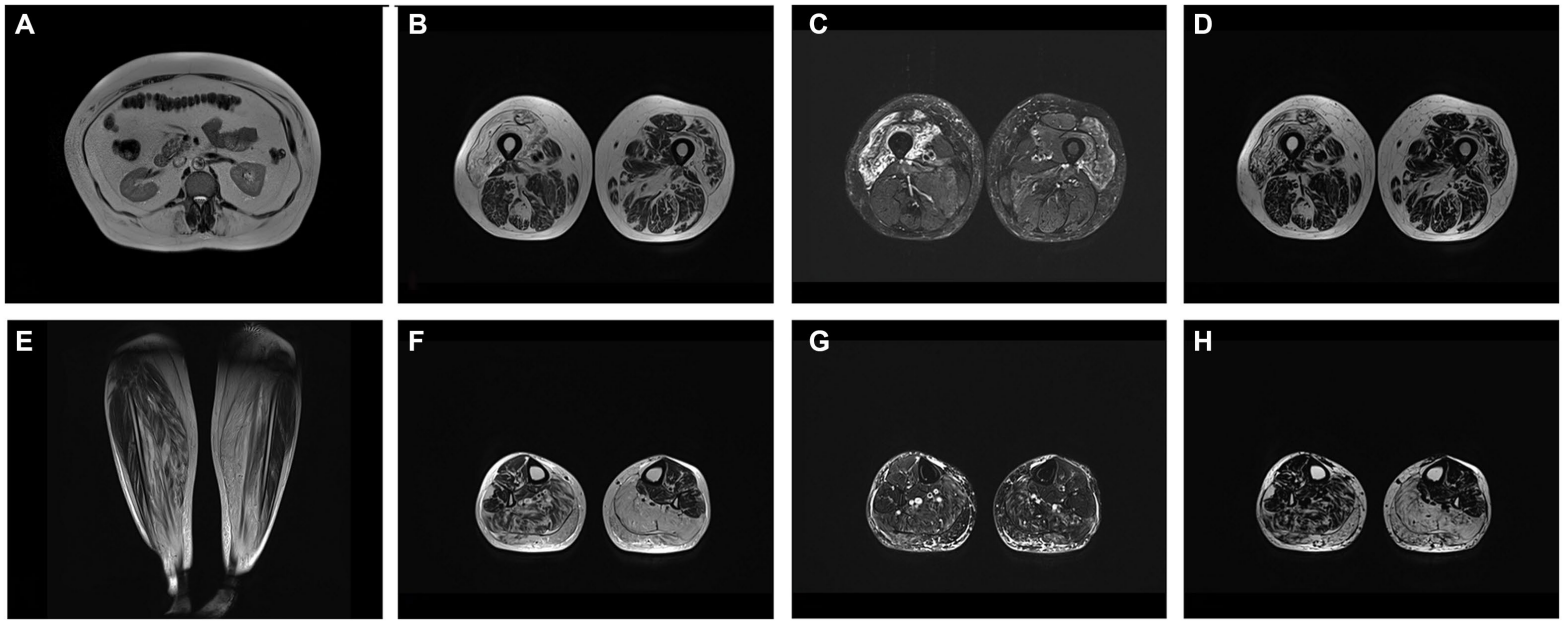
**(A)** T2W axial image at level of L1 showing fatty replacement of abdominal wall and lumbar muscles. Axial at level of middle third of femur T2W **(B)**, T2 DIXON WATER **(C)** and FAT **(D)** images showing mm. vastus intermedius and medialis edema with fatty replacement. Coronal T2W **(E)**, axial at level of proximal third of calf T2W **(F)**, T2 DIXON WATER **(G)** and FAT **(H)** images showing fatty replacement of muscle in mm. soleus and gastrocnemius (more prominent on left side).

Whole body MRI showed fatty replacement, predominantly affecting right vastus intermedius and medialis, gastrocnemius and soleus in calf, abdomen wall and lumbar muscles (Figure 1).

Family history revealed a similar phenotype of the patient’s father and paternal uncle. The uncle had difficulty walking and experienced muscle weakness from the age of 40, however he died at 59.

The patient’s father is a 73-year-old male. At the age of 60, he started to struggle climbing stairs, and he suffered from frequent falls. At the age of 68, he had difficulty walking and started using crutches. He also has difficulty standing up from sitting and lying positions. ENMG examination revealed signs of partial axonal degeneration of the motor nerves in the lower limbs. Myopathic patterns were recorded in all muscles of the lower limbs (especially the quadriceps femoris) and the proximal muscles of the upper limbs. His CK level was 119 U/L.

Next-generation sequencing analysis of the proband revealed a previously reported pathogenic heterozygous variant NM_007126.5:c.277C > T (NP_009057.1:p.(Arg93Cys)) in exon 3 of the *VCP* gene. Based on the guidelines of ACMG/AMP for interpretation of sequence variants, it is classified as pathogenic (categories: PP5, PP3, PM1, PM5, and PM2) (12). Segregation analysis showed that the detected variant is inherited from the affected father. The detailed sequencing and analysis methods have previously been described (13).

## 3 Discussion

Myopathy is the most frequent manifestation of VCP related disease affecting around 90% of patients with pathogenic variants in *VCP* gene (1, 2). It is characterized by slowly progressing weakness and atrophy of proximal and distal upper and lower limb muscles, initially involving shoulder and pelvic muscles (1, 2, 14). Some of the other symptoms include axial muscle weakness, scapular winging, respiratory impairment, lower motor neuron sings, dysautonomia, dysphagia (1). Patients often have abnormal gait, lordotic stance, experience difficulty climbing stairs, raising arms (14, 15). Our patient presented with typical symptoms: weakness of proximal and distal limbs, atrophied shoulder and pelvic girdle muscles, wide based gait, lordotic posture. Both the patient and his father experienced difficulties in daily activities such as climbing up the stairs, standing up from a sitting position. However, the patient’s father is unable to move around without the crutches showing the progression of the disease.

An electromyographic (EMG) examination showed a myopathic pattern mainly in patient’s proximal limb muscles and in all leg muscles and proximal arm muscles in patient’s father. Myopathic pattern in EMG is characteristic of IBM. Purely myopathic pattern is observed in 31–47% of patients in literature. 11–21% of patients have neurogenic and 14–20% mixed myopathic and neurogenic pattern (1, 3). The patient‘s father showed signs of partial axonal degeneration of the motor nerves in the legs. Motor neuron involvement was reported in 25% of patients in Schiava et al. cohort. Of those, almost a half showed exclusively lower motor neuron signs (1).

It is worth mentioning that while patients with VCP related myopathy usually exhibit normal or slightly increased CK levels (2–4), the CK levels of our proband are higher than what is typically observed. However, there have been some reported cases of elevated CK levels in individuals with certain *VCP* gene variants, such as homozygous p.Arg159His variant with early disease onset at 29 years (16), or the newly described case of a heterozygous variant of c.760A > T with CK levels >2,378 (17). The patient’s relatively early age of disease onset and the aggressive progression of their symptoms are also noteworthy. While the mean age of symptoms onset for VCP related disease is around 43 years (2, 3), there can be a significant variation depending on the specific variant and other factors. Patients with heterozygous c.277C > T variant experience first symptoms later than our patient (1). Similarly, patient’s uncle had difficulty walking at the age of 40. In comparison, patient’s father experienced the onset of symptoms at the age of 60, which has been observed in several cases with c.277C > T variant (18, 19). As a result, the combination of early onset and high CK levels might suggest a more severe form of the disease in this patient.

Human Gene Mutation Database (HGMD) reports 71 variants of *VCP* gene, 66 of those are missense/nonsense (20). In Varsome database 89 of the reported *VCP* gene variants were identified as pathogenic or likely pathogenic (21). Although genotype–phenotype correlations are not yet well defined a few studies have identified some correlations between phenotype and specific variants (2, 3). In Schiava et al. study variant c.277C > T was associated with older age of disease onset compared to c.464G > A. Median age for c.277C > T variant was 52.3 ± 5.6 years (1). Table 2 summarizes clinical findings of reported patients with c.277C > T. Of the reported cases in literature with c.277C > T variant, the lowest age of symptom manifestation was 42 years (23). In the reported cases with c.277C > T variant, most patients have myopathy together with FTD or PDB. In comparison, only one case was reported with all 3 manifestations of the disease and only one case where FTD was the only manifestation of the disease (19). CK in most of the reported cases was slightly above normal. Most reported patients did not lose ambulation, but some have difficulty moving (25, 26). Both proximal and distal muscles were affected in reported cases with c.277C > T variant (1).

**Table 2:** Clinical findings of patients with c.277C > T variant of *VCP* gene found in literature.

Reference	*N*	Type	Age of onset	Age at follow up	CK (U/L)	ALP (IU/L)	Wheelchair use	Muscle weakness (onset)
Schiava et al. (1)	17	Muscle weakness – 94% FTD – 24% PDB – 44%	52.3 ± 5.6	64.2 ± 5.3	NA	NA	20%	Proximal UL 80%; Proximal LL 90%; Distal UL and LL 73%
Mengel et al. (22)	1	IBM + cognitive impairment	50	74	288	NA	NA	Distal > proximal
Figueroa-Bonaparte et al. (23)	1	IBM + cognitive impairment	42	56	400	high	No	Distal LL
	1	IBM + cognitive impairment	48	67	normal	normal	No	Distal LL > UL
Mehta et al. (3)	2	IBM + PDB IBM + FTD	60	NA	370	NA	NA	NA
Shi et al. (24)	1	IBM	58	70	286	NA	NA	Proximal>distal
Fanganiello et al. (25)	1	IBM + PDB	55	62	285–572	NA	No, has difficulties moving	Proximal>distal
Krause et al. (26)	1	IBM + FTD	55	74	115	154	No, requires assistance	Distal>proximal
Guyant- Maréchal et al. (19)	1	FTD	60	61	NA	NA	NA	Proximal and distal muscles, absent in muscles of face, tongue, and scapular fixators
	1	PBD + FTD	55	60	NA	NA	NA	
	1	IBM + PDB + FTD	48	68	NA	NA	NA	
	1	IBM + FTD	44	49	NA	NA	NA	
Present study proband	1	IBM	38	49	1,237–1,309	56	No, uses crutches	Proximal UL, proximal and distal LL
Present study affected father	1	IBM	60	69	92–119	87	No, uses crutches	Proximal UL, proximal and distal LL

N, number of patients; NA, not available; FTD, frontotemporal dementia; IBM, inclusion body myopathy; PDB, Paget disease of bone; CK, creatine kinase; ALP, alkaline phosphatase; UL, upper limb; LL, lower limb.

As muscle weakness progresses, especially in the lower limbs, patients eventually lose ambulation. In Schiava et al. study after 8.5 years 23% of the patients were no longer ambulant (1). In Figueroa-Bonaparte et al. study mean time to lose ambulation was 13.37 ± 6.6 years (23). Over time muscle weakness involves respiratory and cardiac muscles leading to death from respiratory or cardiac failure (1, 3). Schiava et al. identified forced vital capacity (FCV) below 50% as the major risk factor associated with loss of ambulation and FCV below 70% with higher risk of death (1). Patients usually die in their 60s 15–20 years after the onset of the disease (1, 3).

This study adds to previous evidence demonstrating a summary of clinical findings of reported patients with c.277C > T variant in *VCP* gene.

## Data availability statement

The original contributions presented in the study are included in the article/supplementary material, further inquiries can be directed to the corresponding author.

## Ethics statement

Written informed consent to participate in this study was not required from the participants or the participants’ legal guardians/next of kin in accordance with the national legislation and the institutional requirements. Written informed consent was obtained from the individual(s) for the publication of any potentially identifiable images or data included in this article.

## Author contributions

GA: Conceptualization, Writing – original draft. RV: Data curation, Writing – review & editing. VA: Data curation, Writing – review & editing. BB: Conceptualization, Data curation, Supervision, Writing – review & editing.

## References

[1] Schiava M Ikenaga C Villar-Quiles RN Caballero-Ávila M Topf A Nishino I. Genotype–phenotype correlations in valosin-containing protein disease: a retrospective multicenter study. Neurol Neurosurg Psychiatry. (2022) 93:1099–111. doi: 10.1136/jnnp-2022-328921, PMID: 35896379 PMC9880250

[2] Al-Obeidi E Al-Tahan S Surampalli A Goyal N Wang AK Hermann A. Genotype-phenotype study in patients with valosin-containing protein mutations associated with multisystem proteinopathy. Clin Genet. (2018) 93:119–25. doi: 10.1111/cge.13095, PMID: 28692196 PMC5739971

[3] Mehta SG Khare M Ramani R Watts GDJ Simon M Osann KE. Genotype-phenotype studies of VCP-associated inclusion body myopathy with paget disease of bone and/or frontotemporal dementia. Clin Genet. (2013) 83:422–31. doi: 10.1111/cge.12000, PMID: 22909335 PMC3618576

[4] Wang SC Smith CD Lombardo DM Kimonis V. Characteristics of VCP mutation-associated cardiomyopathy. Neuromuscul Disord. (2021) 31:701–5. doi: 10.1016/j.nmd.2021.06.005, PMID: 34244020

[5] Gonzalez MA Feely SM Speziani F Strickland AV Danzi M Bacon C. A novel mutation in VCP causes Charcot-Marie-tooth type 2 disease. Brain. (2014) 137:2897–902. doi: 10.1093/brain/awu224, PMID: 25125609 PMC4208462

[6] Souza PVS Bortholin T Dias RB Chieia MAT Burlin S Naylor FGM. New genetic causes for complex hereditary spastic paraplegia. J Neurol Sci. (2017) 379:283–92. doi: 10.1016/j.jns.2017.06.019 28716262

[7] Meyer H Weihl CC. The VCP/p97 system at a glance: connecting cellular function to disease pathogenesis. J Cell Sci. (2014) 127:3877–83. doi: 10.1242/jcs.093831, PMID: 25146396 PMC4163641

[8] van den Boom J Meyer H. VCP/p97-mediated unfolding as a principle in protein homeostasis and signaling. Mol Cell. (2018) 69:182–94. doi: 10.1016/j.molcel.2017.10.028 29153394

[9] Ferrari V Cristofani R Tedesco B Crippa V Chierichetti M Casarotto E. Valosin containing protein (VCP): a multistep regulator of autophagy. Int J Mol Sci. (2022) 23:1939. doi: 10.3390/ijms23041939, PMID: 35216053 PMC8878954

[10] Poksay KS Madden DT Peter AK Niazi K Banwait S Crippen D. Valosin-containing protein gene mutations: cellular phenotypes relevant to neurodegeneration. J Mol Neurosci. (2011) 44:91–102. doi: 10.1007/s12031-010-9489-8, PMID: 21249466 PMC3084943

[11] Watts GDJ Wymer J Kovach MJ Mehta SG Mumm S Darvish D. Inclusion body myopathy associated with Paget disease of bone and frontotemporal dementia is caused by mutant valosin-containing protein. Nat Genet. (2004) 36:377–81. doi: 10.1038/ng1332, PMID: 15034582

[12] Richards S Aziz N Bale S Bick D Das S Gastier-Foster J. Standards and guidelines for the interpretation of sequence variants: a joint consensus recommendation of the American College of Medical Genetics and Genomics and the Association for Molecular Pathology. Genet Med. (2015) 17:405–24. doi: 10.1038/gim.2015.30, PMID: 25741868 PMC4544753

[13] Siavrienė E Petraitytė G Burnytė B Morkūnienė A Mikštienė V Rančelis T. Compound heterozygous c.598_612del and c.1746-20C > G CAPN3 genotype cause autosomal recessive limb-girdle muscular dystrophy-1: a case report. BMC Musculoskelet Disord. (2021) 22:1020. doi: 10.1186/s12891-021-04920-3, PMID: 34863162 PMC8645139

[14] Kimonis V. Inclusion body myopathy with Paget disease of bone and/or frontotemporal dementia In: Adam MP Mirzaa GM Pagon RA, editors. GeneReviews®. Seattle: University of Washington (1993)

[15] Kimonis VE Fulchiero E Vesa J Watts G. VCP disease associated with myopathy, Paget disease of bone and frontotemporal dementia: review of a unique disorder. Biochim Biophys Acta Mol basis Dis. (2008) 1782:744–8. doi: 10.1016/j.bbadis.2008.09.003, PMID: 18845250

[16] De Ridder W Azmi A Clemen CS Eichinger L Hofmann A Schröder R. Multisystem proteinopathy due to a homozygous p.Arg159His VCP mutation: a tale of the unexpected. Neurology. (2020) 94:e785–96. doi: 10.1212/WNL.0000000000008763, PMID: 31848255

[17] Columbres RCA Chin Y Pratti S Quinn C Gonzalez-Cuyar LF Weiss M. Novel variants in the VCP gene causing multisystem Proteinopathy 1. Genes. (2023) 14:676. doi: 10.3390/genes14030676, PMID: 36980948 PMC10048343

[18] Al-Tahan S Al-Obeidi E Yoshioka H Lakatos A Weiss L Grafe M. Novel valosin-containing protein mutations associated with multisystem proteinopathy. Neuromuscul Disord. (2018) 28:491–501. doi: 10.1016/j.nmd.2018.04.007, PMID: 29754758

[19] Guyant-Maréchal L Laquerrière A Duyckaerts C Dumanchin C Bou J Dugny F. Valosin-containing protein gene mutations. Neurology. (2006) 67:644–51. doi: 10.1212/01.wnl.0000225184.14578.d3, PMID: 16790606

[20] Stenson PD Mort M Ball EV Chapman M Evans K Azevedo L. The human gene mutation database (HGMD®): optimizing its use in a clinical diagnostic or research setting. Hum Genet. (2020) 139:1197–207. doi: 10.1007/s00439-020-02199-3, PMID: 32596782 PMC7497289

[21] Kopanos C Tsiolkas V Kouris A Chapple CE Albarca Aguilera M Meyer R. VarSome: the human genomic variant search engine. Bioinformatics. (2019) 35:1978–80. doi: 10.1093/bioinformatics/bty897, PMID: 30376034 PMC6546127

[22] Mengel D Librizzi D Schoser B Gläser D Clemen CS Dodel R. Inclusion body myopathy, paget’s disease, and Fronto-temporal dementia: a VCP-related multi-systemic proteinopathy. Fortschr Neurol Psychiatr. (2018) 86:434–8. doi: 10.1055/s-0044-101033, PMID: 30029282

[23] Figueroa-Bonaparte S Hudson J Barresi R Polvikoski T Williams T Töpf A. Letter: mutational spectrum and phenotypic variability of VCP-related neurological disease in the UK. J Neurol Neurosurg Psychiatry. (2016) 87:680–1. doi: 10.1136/jnnp-2015-310362, PMID: 26105173 PMC4893144

[24] Shi Z Hayashi YK Mitsuhashi S Goto K Kaneda D Choi YC. Characterization of the Asian myopathy patients with VCP mutations. Eur J Neurol. (2012) 19:501–9. doi: 10.1111/j.1468-1331.2011.03575.x 22040362

[25] Fanganiello RD Kimonis VE Côrte CC Nitrini R Passos-Bueno MR. A Brazilian family with hereditary inclusion body myopathy associated with paget disease of bone and frontotemporal dementia. Braz J Med Biol Res. (2011) 44:374–80. doi: 10.1590/S0100-879X2011007500028, PMID: 21412659

[26] Krause S Göhringer T Walter MC Schoser BGH Reilich P Linn J. Brain imaging and neuropsychology in late-onset dementia due to a novel mutation (R93C) of valosin-containing protein. Clin Neuropathol. (2007) 26:232–40. doi: 10.5414/npp26232, PMID: 17907600

